# The interplay between multisite pain and insomnia on the risk of anxiety and depression: the HUNT study

**DOI:** 10.1186/s12888-022-03762-0

**Published:** 2022-02-16

**Authors:** Anna Marcuzzi, Eivind Schjelderup Skarpsno, Tom Ivar Lund Nilsen, Paul Jarle Mork

**Affiliations:** 1 Department of Public Health and Nursing, Norwegian University of Science and Technology (NTNU), 7491 Trondheim, Norway; 2 Department of Neurology and Clinical Neurophysiology, St. Olavs Hospital, Trondheim, Norway; 3 Clinic of Anaesthesia and Intensive Care, St. Olavs Hospital, Trondheim, Norway

**Keywords:** Musculoskeletal pain, Insomnia, Anxiety, Depression, Mental health, Prospective

## Abstract

**Background:**

Chronic musculoskeletal pain and insomnia frequently co-occur and are known independent risk factors for anxiety and depression. However, the interplay between these two conditions on the risk of anxiety and depression has not been explored.

**Methods:**

A population-based prospective study of 18,301 adults in the Norwegian HUNT Study without anxiety or depression at baseline (2006–2008). We calculated adjusted risk ratios (RRs) with 95% confidence intervals (CIs) for anxiety and/or depression at follow-up (2017–2019), associated with i) number of chronic pain sites, and ii) chronic pain and insomnia symptoms jointly.

**Results:**

At follow-up, 2155 (11.8%) participants reported anxiety and/or depression. The number of pain sites was positively associated with risk of anxiety and/or depression (P_trend_, < 0.001). Compared to people without chronic pain and insomnia symptoms, people with ≥5 pain sites and no insomnia symptoms had a RR of 1.52 (95% CI: 1.28 to 1.81) for anxiety and/or depression, those with no chronic pain but with insomnia had a RR of 1.78 (95% CI: 1.33 to 2.38), whereas the RR among people with both ≥5 pain sites and insomnia was 2.42 (95% CI: 1.85 to 3.16). We observed no synergistic effect above additivity for the combination of ≥5 pain sites and insomnia on risk of anxiety and/or depression.

**Conclusions:**

This study shows that people with multisite chronic pain who also suffer from insomnia are at a particularly high risk for anxiety and/or depression, suggesting that insomnia symptoms are important contributors to the association between multisite pain and common mental health problems.

**Supplementary Information:**

The online version contains supplementary material available at 10.1186/s12888-022-03762-0.

## Introduction

Anxiety and depression are highly prevalent conditions and significant contributors to the disability burden worldwide [1]. It is estimated that 27% of the adult European population is affected by a mental disorder every year, with anxiety and depression being the most common conditions [2]. The adverse impact of anxiety and depression on individuals and society [3, 4] advocates for research efforts aimed at identifying modifiable risk factors that can be targeted by preventive strategies.

Population-based research has shown that chronic pain is associated with increased risk of depression [5–10] and anxiety [9–11], and this effect seems to be more pronounced when higher pain severity or activity interfering pain was reported. Additionally, changes in musculoskeletal pain severity has been associated with subsequent depression severity in clinical samples [12]. Musculoskeletal pain most often occurs in more than one body site [13], and pain at multiple sites is shown to be a marker of poor pain-related health outcomes [14]. One study found a positive association between the number of pain sites and the onset of anxiety and depression [11], suggesting that multisite pain is also an important risk factor for mental health problems.

Moreover, it is well established that insomnia is a strong risk factor for anxiety and depression [15, 16]. Insomnia symptoms are frequent in people with musculoskeletal pain [17–19] and these conditions appear to be bi-directionally related, where pain increases the risk of insomnia [20] and vice versa [21]. The pathways linking insomnia and musculoskeletal pain with mood disorders are thought to be complex and include altered immune responses [22–25], neurobiological changes such as dysregulations in monoamine levels [26], alteration in brain regions involved in emotional processing [27], as well as psychological stressors such as perceived stress, loss of control, maladaptive coping, and social support [28]. Thus, musculoskeletal pain and insomnia are interrelated, and it is conceivable that people who suffer from both conditions may have a particularly high risk of anxiety and depression. However, the effect of this interplay between chronic musculoskeletal pain and insomnia on the risk of anxiety and depression is not known.

The aim of the current study is therefore to examine the prospective association between number of chronic musculoskeletal pain sites and the risk of anxiety and depression and to explore the potentially modifying effect of insomnia on this association. We hypothesized that insomnia symptoms may exaggerate the association between multisite pain and risk of anxiety and depression.

## Methods

### Study population

The Norwegian HUNT Study is a longitudinal population-based cohort study carried out in the Nord-Trøndelag County in Norway [29]. The current study was based on data from the third (HUNT3, 2006–2008) and fourth (HUNT4, 2017–2019) surveys of the HUNT Study. All inhabitants aged 20 years or more residing in Nord-Trøndelag county in Norway were invited to participate in the HUNT Study. Of 93,860 eligible participants in HUNT3, 50,807 (54%) accepted the invitation to participate, while in HUNT4, a total of 103,736 participants were invited, and 56,078 (54%) accepted the invitation. A detailed description of participation rates, questionnaires, and clinical examinations can be found at https://www.ntnu.edu/hunt.

For this prospective study we selected the 33,819 people who participated at both baseline in 2006–2008 (HUNT3) and follow-up in 2017–2019 (HUNT4). Of these, we excluded 6628 people who had incomplete information about depression and/or anxiety, musculoskeletal pain, and insomnia at baseline. Furthermore, we excluded 4665 people who reported anxiety and/or depression at baseline. Of the remaining 22,526 participants, 18,301 had complete information about depression and anxiety at follow-up in 2017–2019 and were included in the analysis.

### Chronic musculoskeletal pain

The Standardised Nordic Questionnaire [30] was used to retrieve information about musculoskeletal pain. Participants who reported having chronic musculoskeletal pain ticking ‘yes’ on the following question “During the last year, have you had pain and/or stiffness in muscles or joints that lasted for at least 3 consecutive months?” were asked to indicate the affected body area(s), i.e., neck, shoulders, upper back, elbows, low back, hips, wrists/hands, knees, and ankles/feet. Participants were categorised based on the number of pain sites, regardless of the pain location, as ‘no chronic pain’, ‘1-2 pain sites’, ‘3-4 pain sites’ and ‘≥5 pain sites.’ These categories were adapted based on the classification of pain reported in Coggon et al. [13] to distinguish between limited pain (i.e., 1–2 pain sites) and extensive pain (i.e., ≥5 pain sites).

### Insomnia symptoms

Insomnia symptoms were assessed by four questions which are part of the sleep related questions in HUNT (sHUNT-Q) [31]: 1) “How often during the last 3 months have you had difficulty falling asleep at night?”, 2) “How often during the last 3 months have you woken up repeatedly during the night?”, 3) “How often during the last 3 months have you woken up too early and could not get back to sleep?” and 4) “How often during the last 3 months have you felt sleepy during the day?” with three response options ‘never/seldom’, ‘sometimes’ and ‘several times a week’. These self-reported questions have shown acceptable validity with kappa values ranging from 0.35 to 0.44 when compared with interviews performed by clinician [31]. In addition, participants were classified with insomnia if they answered ‘several times a week’ for at least one of questions 1–3 *and* ‘several times a week’ for question 4. This classification approximates insomnia diagnosis according to the current international classification of sleep disorders (ICSD-3) [32]. Participants who did not fulfill the insomnia diagnostic classification, but who reported any insomnia symptoms, i.e., answered ‘several times a week’ to at least one of questions 1–3 *or* question 4, were classified as having sub-threshold insomnia.

### Anxiety and depression

The Hospital Anxiety and Depression Scale (HADS) is a self-reported scale encompassing 14 items scored on a 4-point Likert scale, of which 7 items assess depression (HADS-D) and 7 items assess anxiety (HADS-A). Scores for each subscale range from 0 to 21 with higher scores indicating higher depression and anxiety levels. A cut-off score of ≥8 for each subscale was chosen to indicate the presence of depression or anxiety, respectively [33]. We reported three outcomes: anxiety and/or depression defined by a score of at least ≥8 on either scale; anxiety defined by HADS-A score ≥ 8; depression defined by HADS-D score ≥ 8. Additionally, the anxiety *and* depression outcome defined by a score of at least ≥8 on both scales was used in an exploratory analysis.

### Assessment of covariate variables

All possible confounders were assessed at baseline (HUNT3, 2006–2008). Body mass index (BMI) was calculated as weight divided by the square of height (kg/m^2^) using standardized measurements of height (to the nearest centimeter) and weight (to the nearest half kilogram) from the clinical examination. Leisure time physical activity was assessed by three questions on frequency (0, < 1, 1, 2–3 and ≥ 4 times per week), duration (< 15, 15–30, 31–60 and > 60 min) and intensity (light, moderate, vigorous) of physical exercise per week [34]. Participants who reported exercising less than once a week were categorized as inactive. For participants who reported exercising at least once a week, a summary score was constructed by giving equal weight to each measure of frequency, duration, and intensity according to the following equation: 1/5 x frequency + 1/4 x duration + 1/3 x intensity [35]. The resulting summary scores ranged from 1.18 to 3.00 and cutoff values of 1.63 and 2.02 were derived based on its distribution. Participants were then classified into 3 exercise groups: low activity (≤1.63), moderate activity (1.64–2.02) and high activity (≥2.02). Smoking status was assessed by several questions related to current and past cigarette smoking and categorized as: ‘never smoked’, ‘former or occasional smoker’ and ‘current smoker’. Alcohol consumption was assessed by the CAGE (Cut down, Annoyed, Guilty, Eye-opener) questionnaire [36] using a cutoff score ≥ 2 to indicate possible alcohol abuse. To assess comorbid conditions participants were asked about current and previous disease (cardiovascular disease, diabetes, cancer, rheumatic disease, or degenerative joint disease).

### Statistical analysis

A modified Poisson regression model was used to estimate risk ratios (RR) with 95% confidence intervals (CIs) for anxiety and depression, predominant anxiety, and predominant depression at follow-up in 2017–2019, associated with chronic musculoskeletal pain at baseline in 2006–2008. All CIs were estimated using robust variance. People without chronic musculoskeletal pain were used as reference group for all analyses. *P*-values from test of linear trend (P_trend_) were obtained from analyses treating number of pain sites as an ordinal variable in the regression model. All associations were adjusted for age (continuous), gender (women, men), BMI (continuous), leisure time physical activity (inactive, low, moderate, high), alcohol consumption (no alcohol abuse, possible alcohol abuse), smoking (never, former, current smoker). BMI (0.1%), physical activity (1.9%), smoking (2%), alcohol consumption (10.8%) and comorbidities (4.1%) had missing data which were imputed (20 imputations) using all variables in the main analysis as predictors in the imputation model.

To estimate the joint effect of chronic musculoskeletal pain and insomnia symptoms on the risk of mental health problems at follow-up, people without chronic musculoskeletal pain and no insomnia symptoms were used as the reference group. Anxiety and/or depression combined was used as outcome in this analysis. Due to the limited number of cases, the other outcomes were reported only for the main analysis. The relative excess risk due to interaction (RERI) was calculated to investigate the potential effect modification between the variables as departure from additive effect. This was calculated along with 95% CIs using the following equation: RR_insomnia and ≥ 5 pain sites_ – RR_no insomnia and ≥ 5 pain sites_ – RR_insomnia and pain-free_ + 1 [37]. A RERI > 0 indicates a synergistic effect beyond an additive effect.

We performed two supplementary analyses to assess the influence of potential bias due to reverse causation. First, we repeated the main analyses excluding people who reported having ever sought help for any mental health problem. Second, we excluded people who reported strong or very strong physical pain over the last month within the group without chronic musculoskeletal pain. In this latter analysis, we aimed to exclude people who could have developed chronic pain following an acute pain episode and thereby influenced the estimated risk of anxiety and depression at follow-up despite being classified without chronic pain at baseline. Additionally, we excluded people who reported fibromyalgia as a greater proportion of these people are expected to report ≥5 pain sites. Moreover, fibromyalgia is commonly characterized by sleep problems, which might potentially influence the estimates of the joint effect of pain and insomnia on the risk of anxiety and depression. Finally, we repeated the main analyses adjusting for chronic conditions that may confound the association between chronic pain, insomnia and anxiety and depression (i.e., cardiovascular disease, diabetes, cancer, rheumatic disease, or degenerative joint disease). These factors were not part of the main analysis since they could potentially induce collider bias.

All statistical analyses were performed using Stata for Windows, version 16.0 (StataCorp LP, College Station, Texas).

## Results

Table 1 presents baseline characteristics of the study population, stratified by number of musculoskeletal pain sites. The proportion of participants reporting any chronic pain at baseline was 48.7%. Among them, 46.6% reported 1–2 pain sites, 32.1% reported 3–4 pain sites, and 21.2% reported ≥5 pain sites. At follow-up, 2155 (11.8%) participants reported anxiety and/or depression. Of these, 1615 (8.8%) reported anxiety and 926 (5.1%) reported depression. The number of participants reporting both anxiety and depression was 386.

**Table 1:** Baseline characteristics of the study population stratified by number of chronic musculoskeletal pain sites

Variables	No chronic pain	Number of chronic pain sites		
		1–2 pain sites	3–4 pain sites	≥5 pain sites
Participants, no.	9380	4161	2865	1895
Age, mean (SD) [range], years	50.2 (13.6) [19–89]	52.9 (12.5) [19–87]	54.4 (11.7) [19–85]	56.2 (10.5) [21–90]
Females, % (no.)	53.4 (5011)	53.6 (2231)	65.9 (1888)	74.1 (1404)
Body mass index, mean (SD), kg/m^2^	26.6 (4.0)	27.1 (4.1)	27.8 (4.4)	28.3 (4.6)
Inactive, % (no.)^a^	16.1 (1.514)	17.8 (740)	17.4 (500)	18.9 (358)
Insomnia, % (no.)^b^	2.4 (230)	3.5 (146)	7.1 (205)	11.0 (210)
Current smoker, % (no.)	10.8 (1.011)	13.1 (544)	15.5 (444)	19.0 (360)
Possible alcohol abuse, % (no.)^c^	6.7 (625)	6.8 (285)	5.9 (168)	5.2 (98)
Comorbid conditions, % (no.)^d^	14.3 (1344)	25.9 (1078)	37.8 (1082)	58.6 (1110)
* Cardiovascular disease, % (no.)*	6.1 (574)	7.4 (307)	8.7 (250)	11.9 (226)
* Diabetes, % (no.)*	2.7 (256)	3.0 (125)	3.8 (110)	5.8 (110)
* Rheumatic/degenerative joint disease, % (no.)*	4.1 (380)	15.7 (653)	27.4 (784)	46.0 (871)
* Cancer, % (no.)*	3.8 (358)	4.6 (190)	5.4 (154)	6.4 (122)

*SD* standard deviation

^a^Physical activity less than once per week; ^b^At least one nighttime insomnia symptom (‘difficulty falling asleep’, ‘difficulty maintaining sleep’, or ‘waking up too early’) accompanied by daytime sleepiness; ^c^Score ≥ 2 on the CAGE questionnaire; ^d^Any of the following: cardiovascular disease, diabetes, cancer, rheumatic disease, degenerative joint disease

Table 2 shows the association between the number of chronic pain sites and the risk of anxiety and/or depression at follow-up. Compared to the reference group of people without chronic pain, those who reported 1–2 pain sites, 3–4 pain sites and ≥ 5 pain sites had RRs for anxiety and/or depression of 1.13 (95% CI: 1.02 to 1.25), 1.34 (95% CI: 1.19 to 1.50) and 1.54 (95% CI: 1.36 to 1.75), respectively (P_trend_, < 0.001). When examining anxiety and depression separately, people with chronic pain at 1–2 pain sites, 3–4 pain sites and ≥ 5 pain sites had RRs for anxiety of 1.13 (95% CI: 1.00 to 1.28), 1.38 (95% CI: 1.21 to 1.57) and 1.60 (95% CI: 1.38 to 1.85), respectively. The corresponding RRs for depression were 1.30 (95% CI: 1.11 to 1.52), 1.29 (95% CI: 1.08 to 1.54) and 1.45 (95% CI: 1.18 to 1.76). Finally, for the exploratory analysis on anxiety *and* depression outcome, people with chronic pain at 1–2 pain sites, 3–4 pain sites and ≥ 5 pain sites had RRs of 1.54 (95% CI: 1.21 to 1.96), 1.34 (95% CI: 1.00 to 1.79) and 1.41 (95% CI: 1.01 to 1.97) compared to the reference group of people without chronic pain.

**Table 2:** Risk of anxiety and depression at follow-up associated with number of chronic pain sites at baseline

	Anxiety and/or depression^a^				Anxiety^b^			Depression^c^		
Chronic pain variables	No. of persons	No. of cases	Age-adjusted RR^d^	Multi-adjusted, RR^e^ (95% CI)	No. of cases	Age-adjusted RR^d^	Multi-adjusted RR^e^ (95% CI)	No. of cases	Age-adjusted RR^d^	Multi-adjusted RR^e^ (95% CI)
No chronic pain	9380	962	1.00	1.00 (reference)	730	1.00	1.00 (reference)	394	1.00	1.00 (reference)
1–2 pain sites	4161	482	1.14	1.13 (1.02–1.25)	352	1.14	1.13 (1.00–1.28)	240	1.32	1.30 (1.11–1.52)
3–4 pain sites	2865	401	1.39	1.34 (1.19–1.50)	302	1.47	1.38 (1.21–1.57)	166	1.30	1.29 (1.08–1.54)
≥5 pain sites	1895	310	1.64	1.54 (1.36–1.75)	231	1.76	1.60 (1.38–1.85)	126	1.45	1.45 (1.18–1.76)

*CI* confidence interval, *RR* risk ratio

^a^HADS-A ≥ 8 and/or HADS-D ≥ 8;^b^HADS-A ≥ 8 and any HADS-D;^c^HADS-D ≥ 8 and any HADS-A

^d^Adjusted for age (continuous)

^e^Multi-adjusted for age (continuous), sex (women, men), body mass index (continuous), smoking (never, former or occasional, current), alcohol consumption (no alcohol abuse, possible alcohol abuse), physical activity level (inactive, low activity, moderate activity, high activity)

Table 3 shows the joint effect of number of pain sites and insomnia symptoms on the risk of anxiety and/or depression at follow up. Compared to the reference group of people without chronic pain and insomnia symptoms, people with chronic pain at ≥5 pain sites had a RR of 2.42 (95% CI: 1.85 to 3.16) if they had insomnia, a RR of 1.81 (95% CI: 1.51 to 2.17) if they reported subthreshold insomnia, and a RR of 1.52 (95% CI: 1.28 to 1.81) if they reported no insomnia symptoms. Those who reported insomnia, but no chronic pain had an RR of 1.78 (95% CI: 1.33–2.38). There was no evidence for a synergistic effect above additivity for the combination of chronic pain at ≥5 pain sites and insomnia (based on ICSD-3) on risk of anxiety and/or depression (RERI = 0.09; 95% CI: − 0.11 to 0.29).

**Table 3:** Joint effect of number of chronic pain sites and insomnia symptoms on risk of anxiety and depression at follow up

	No insomnia symptoms				Sub-threshold insomnia^a^				Insomnia (ICSD-3)^b^			
Chronic pain variables	No. of persons	No. of cases	Age-adjusted RR	Multi-adjusted, RR^c^ (95% CI)	No. of persons	No. of cases	Age-adjusted RR	Multi-adjusted RR^c^ (95% CI)	No. of persons	No. of cases	Age-adjusted RR	Multi-adjusted RR^c^ (95% CI)
No chronic pain	7653	703	1.00	1.00 (reference)	1497	219	1.60	1.57 (1.36–1.81)	230	40	1.86	1.78 (1.33–2.38)
1–2 pain sites	3063	312	1.12	1.11 (0.98–1.26)	952	149	1.73	1.69 (1.43–1.99)	146	21	1.55	1.48 (0.99–2.21)
3–4 pain sites	1826	223	1.35	1.31 (1.14–1.52)	834	136	1.82	1.74 (1.46–2.06)	205	42	2.24	2.15 (1.62–2.84)
≥5 pain sites	968	139	1.60	1.52 (1.28–1.81)	717	123	1.93	1.81 (1.51–2.17)	210	48	2.56	2.42 (1.85–3.16)

*CI* confidence interval, *RR* risk ratio

^a^At least one of the following symptoms: ‘difficulty falling asleep’, ‘difficulty maintaining sleep’, ‘waking up too early’, ‘daytime sleepiness’

^b^At least one nighttime insomnia symptom (‘difficulty falling asleep’, ‘difficulty maintaining sleep’, or ‘waking up too early’) accompanied by daytime sleepiness

^c^Multi-adjusted for age (continuous), sex (women, men), body mass index (continuous), smoking (never, former or occasional, current), alcohol consumption (no alcohol abuse, possible alcohol abuse), physical activity level (inactive, low activity, moderate activity, high activity)

### Supplementary analyses

First, analyses excluding people who reported to have sought help for any mental health problems at baseline attenuated the strength of the associations by ~ 10–15% compared to the main analysis. Second, excluding people in the pain-free group that reported severe physical pain over the last month at baseline had negligible influence on the estimated associations. Similarly, excluding people with fibromyalgia had negligible influence on the estimated associations. Finally, adjusting for comorbidities had a negligible influence on the estimated associations (Supplementary file).

## Discussion

Our study indicates a positive association between number of chronic musculoskeletal pain sites and risk of anxiety and depression. Further, in people with pain at multiple sites, having insomnia nearly doubles the risk of anxiety and depression compared to not reporting any insomnia symptoms. Overall, these findings suggest that people with multisite chronic pain who also suffer from insomnia are a subgroup at particularly increased risk for developing anxiety and depression.

Previous population-based studies have reported that higher pain severity or pain interference with normal activities increase the risk of anxiety and depression [6, 9–11]. Our finding that people with multisite chronic pain are at higher long-term risk of anxiety and depression is in line with the study by Gerrits et al. [11], showing a 29% increased risk of anxiety and depressive disorders among people who reported an increase in number of pain sites over a 4-year period. However, when examining the risk of both anxiety *and* depression at follow up, our data suggest that the effect is more uniform across number of pain sites. Our study also suggests that multisite chronic pain has a stronger influence on the risk of anxiety compared to depression. People reporting ≥5 pain sites had 60% increased risk of anxiety compared to those without chronic pain, while the corresponding increased risk for depression was 45%. It is important to note that among those reporting anxiety (i.e., HADS-A ≥ 8), 24% also reported depression, and among those reporting depression (i.e., HADS-D ≥ 8) 42% also reported anxiety. In contrast, Gerrits et al. [11] showed a somewhat stronger association between the number of pain sites and depressive disorders compared to anxiety disorders. However, their study only counted pain sites whose pain severity or disability was reported as high, whereas the current study counted all pain sites regardless of their severity. Interestingly, deHeer et al. [9] showed that even low levels of pain severity were strongly associated with increased incidence of anxiety disorders, while higher levels of pain severity and pain interference were more strongly associated with mood disorders. It is therefore possible that the subgroup with multisite pain in our study included people with relatively high levels of function and this might have exerted a smaller influence on the risk of depression. Nonetheless, our findings show that, regardless of its severity, multisite chronic pain is a strong risk factor of poor mental health.

Sleep disturbances have been suggested as a potential pathway linking musculoskeletal pain and mental health [28]. This is partly supported by a prospective community-based study showing that new onset of sleep problems in people with chronic musculoskeletal pain was associated with a three-fold increased risk of depression [38]. Although we cannot make any inference about the causal pathway that may link these conditions to the development of anxiety and depression, our results are important as they show an increased risk of anxiety and depression among people with multisite pain and concurrent insomnia. While there was no statistical evidence of a synergistic effect, we observed that people with ≥3 chronic pain sites and insomnia had a nearly twofold increased risk of anxiety and depression compared to people without insomnia symptoms but with the same number of chronic pain sites. Notably, the risk of anxiety and depression was also heightened for people with chronic musculoskeletal pain reporting subthreshold insomnia, irrespective of the number of pain sites, compared to those without any insomnia symptoms. This finding suggests that intervening on sleep problems in people with chronic musculoskeletal pain before they progress into an advanced insomnia disorder, might be an important strategy to reduce the incidence of anxiety and depression.

### Strengths and limitations

Strengths of the current study include the large population without anxiety or depression at baseline, the prospective design, and the available information on several potential confounders. Furthermore, we had enough cases to assess the association between chronic pain and risk of anxiety and depression separately, and the large sample made it possible to perform analyses of the joint effect of multisite chronic pain and insomnia. Some limitations should be considered in the interpretation of the results. Information was available on frequency of difficulty initiating and maintaining sleep during the last 3 months, which approximate the ICSD-3 criteria for insomnia [32]. However, the response option ‘several times a week’ have been shown to include people who have sleep problems twice a week or more [31], while the insomnia criteria require the symptoms to be present three times or more per week. Moreover, it should be noted that the single items used to assess insomnia had variable levels of reliability [31]. Also, we had no information about the progression of chronic musculoskeletal pain or insomnia symptoms during the follow-up. Similarly, chronic pain and insomnia were assessed only at baseline, and the possible mutual influence between these factors during follow-up could not be considered. Thus, we could not disentangle the temporal interrelation between chronic pain and insomnia or assess if people with both multisite pain and insomnia experienced more disabling pain than those without insomnia but with the same number of pain sites. In addition, no information on prescription pain medications were available to control for as these may have an influence on sleep architecture [39]. Also, we could not discriminate whether musculoskeletal pain was due to cancer or cancer treatment, although only ~ 5% of participants reported a cancer diagnosis. Although we adjusted for several lifestyle and health-related factors, residual confounding due to unknown or unmeasured factors (e.g., genetic or familial factors) influencing both sleep, chronic pain, and anxiety and depression cannot be excluded. Similarly, since anxiety and depression are likely to fluctuate throughout the life course [40], we cannot exclude the possibility that our findings are influenced by reverse causation, e.g., that increased predisposition to anxiety and depression leads to insomnia or chronic pain. However, the supplementary analysis indicated similar associations as in the main analysis when excluding people who reported a history of mental health problems. Finally, incident cases of depression and anxiety were assessed at the follow-up survey among those who were able to and chose to participate in both surveys. Hence, the estimated RRs may be underestimated if participants with chronic pain and/or insomnia symptoms at baseline (HUNT3) were less likely to participate at the follow-up survey (HUNT 4). A previous study showed that people with higher disease burden are less likely to participate in the HUNT Study [41]. However, we looked at the proportions of people within each chronic pain category in those without follow-up data (i.e., not included in the analysis) and found that these were largely similar, ranging from 15.8% in those without pain to 17.6% in those with ≥5 pain sites.

## Conclusions

This study shows that multisite chronic pain represents an independent risk factor for anxiety and depression. Moreover, people with multisite chronic pain who also suffer from insomnia have a markedly higher risk of anxiety and depression compared to people without insomnia but with the same number of chronic pain sites. These findings suggest that insomnia symptoms are important contributors to the association between multisite pain and incidence of common mental health problems.

## Supplementary Information

**Additional file 1.**

## Data Availability

The data that support the findings of this study are available from HUNT Research Centre (https://www.ntnu.edu/hunt) but restrictions apply to the availability of these data, which were used under license for the current study, and so are not publicly available. Data are however available from the co-author “Paul Jarle Mork” (Email ID: paul.mork@ntnu.no) upon reasonable request and with permission of HUNT Research Centre (https://www.ntnu.edu/hunt).

## Abbreviations

RR: Risk Ratio
CI: Confidence Interval
HADS: Hospital Anxiety and Depression Scale
HADS-D: Hospital Anxiety and Depression Scale – Depression Subscale
HADS-A: Hospital Anxiety and Depression Scale – Anxiety Subscale
BMI: Body Mass Index
CAGE: Cut down, Annoyed, Guilty, Eye-opener
RERI: Relative Excess Risk due to Interaction
ICSD-3: International Classification of Sleep Disorders – Third Edition
