# Supplementary material for: The interplay between multisite pain and insomnia on the risk of anxiety and depression: the HUNT study
Source: BMC Psychiatry. 2022 Feb 16;22:124. doi: 10.1186/s12888-022-03762-0 (PMC8851694; doi:10.1186/s12888-022-03762-0)
Supplement: Supplementary file 1 — Additional file 1. [file 12888_2022_3762_MOESM1_ESM.docx]

Table S1. Risk of anxiety and/or depression at follow-up associated with number of chronic pain sites at baseline (excluding people who sought help for any mental health problem)

| Chronic pain variables | Anxiety and/or depression ^a^ | | | |  | Anxiety ^b^ | | |  | | Depression ^c^ | | | |  |
| --- | --- | --- | --- | --- | --- | --- | --- | --- | --- | --- | --- | --- | --- | --- | --- |
|  | No. of persons | No. of cases | Age-adjusted RR^*^ | Multi-adjusted, RR^†^ (95% CI) |  | No. of cases | Age-adjusted RR^*^ | Multi-adjusted RR^†^ (95% CI) | |  | | No. of cases | Age-adjusted RR^*^ | Multi-adjusted RR^†^ (95% CI) | |
|  |  |  |  |  |  |  |  |  |  |  | |  |  |  |  |
| No chronic pain | 8386 | 789 | 1.00 | 1.00 (reference) |  | 592 | 1.00 | 1.00 (reference) | |  | | 321 | 1.00 | 1.00 (reference) | |
| 1-2 pain sites | 3604 | 381 | 1.13 | 1.13 (1.00-1.27) |  | 269 | 1.12 | 1.11 (0.97-1.28) | |  | | 194 | 1.34 | 1.33 (1.12-1.58) | |
| 3-4 pain sites | 2383 | 313 | 1.42 | 1.38 (1.22-1.57) |  | 232 | 1.51 | 1.42 (1.23-1.65) | |  | | 128 | 1.30 | 1.31 (1.07-1.60) | |
| ≥5 pain sites | 1478 | 203 | 1.49 | 1.44 (1.24-1.67) |  | 145 | 1.57 | 1.45 (1.21-1.74) | |  | | 84 | 1.34 | 1.37 (1.08-1.74) | |

CI, confidence interval; RR, risk ratio. ^a^ HADS-A ≥8 and/or HADS-D≥8; ^b^ HADS-A≥8 and any HADS-D; ^c^ HADS-D≥8 and any HADS-A

^*^Adjusted for age (continuous)

^†^ Multi-adjusted for age (continuous), sex (women, men), body mass index (continuous), smoking (never, former or occasional, current), alcohol consumption (no alcohol abuse, possible alcohol abuse), physical activity level (inactive, low activity, moderate activity, high activity)

Table S2. Joint effect of number of chronic pain sites and insomnia symptoms on risk of anxiety and depression at follow up (excluding people who sought help for any mental health problem)

| Chronic pain variables | No insomnia symptoms | | |  | | Any insomnia symptoms ^a^ | | | | |  | | Insomnia (ICSD-3) ^b^ | | | |
| --- | --- | --- | --- | --- | --- | --- | --- | --- | --- | --- | --- | --- | --- | --- | --- | --- |
|  | No. of persons | No. of cases | Multi-adjusted, RR^†^ (95% CI) | |  | | No. of persons | No. of cases | Multi-adjusted RR^†^ (95% CI) |  | | No. of persons | | No. of cases | Multi-adjusted RR^†^ (95% CI) |  |
|  |  |  |  |  |  | |  |  |  |  | |  |  |  |  |  |
| No chronic pain | 6914 | 571 | 1.00 (reference) | |  | 1283 | | 184 | 1.71 (1.47-2.00) |  | | 189 | | 34 | 2.07 (1.51-2.84) |  |
| 1-2 pain sites | 2689 | 248 | 1.13 (0.98-1.30) | |  | 810 | | 120 | 1.79 (1.49-2.15) |  | | 105 | | 13 | 1.45 (0.86-2.43) |  |
| 3-4 pain sites | 1537 | 174 | 1.37 (1.16-1.61) | |  | 688 | | 108 | 1.88 (1.55-2.28) |  | | 158 | | 31 | 2.34 (1.69-3.24) |  |
| ≥5 pain sites | 779 | 94 | 1.45 (1.18-1.78) | |  | 540 | | 76 | 1.69 (1.34-2.12) |  | | 159 | | 33 | 2.47 (1.78-3.40) |  |

CI, confidence interval; RR, risk ratio.

^a^ Any of the following symptoms: ‘difficulty falling asleep’, ‘difficulty maintaining sleep’, ‘waking up too early’, ‘daytime sleepiness’

^b^ At least one nighttime insomnia symptom (‘difficulty falling asleep’, ‘difficulty maintaining sleep’, or ‘waking up too early’) accompanied by daytime sleepiness

^†^ Multi-adjusted for age (continuous), sex (women, men), body mass index (continuous), smoking (never, former or occasional, current), alcohol consumption (no alcohol abuse, possible alcohol abuse), physical activity level (inactive, low activity, moderate activity, high activity)

Table S3. Risk of anxiety and/or depression at follow-up associated with number of chronic pain sites at baseline (excluding people reporting physical pain (strong or very strong) over the last month among those without chronic pain)

| Chronic pain variables | Anxiety and/or depression ^a^ | | | |  | Anxiety ^b^ | | |  | | Depression ^c^ | | | |  |
| --- | --- | --- | --- | --- | --- | --- | --- | --- | --- | --- | --- | --- | --- | --- | --- |
|  | No. of persons | No. of cases | Age-adjusted RR^*^ | Multi-adjusted, RR^†^ (95% CI) |  | No. of cases | Age-adjusted RR^*^ | Multi-adjusted RR^†^ (95% CI) | |  | | No. of cases | Age-adjusted RR^*^ | Multi-adjusted RR^†^ (95% CI) | |
|  |  |  |  |  |  |  |  |  |  |  | |  |  |  |  |
| No chronic pain | 8427 | 867 | 1.00 | 1.00 (reference) |  | 666 | 1.00 | 1.00 (reference) | |  | | 350 | 1.00 | 1.00 (reference) | |
| 1-2 pain sites | 4161 | 482 | 1.14 | 1.12 (1.01-1.25) |  | 352 | 1.14 | 1.12 (0.99-1.27) | |  | | 240 | 1.32 | 1.29 (1.10-1.52) | |
| 3-4 pain sites | 2865 | 401 | 1.39 | 1.31 (1.17-1.47) |  | 302 | 1.46 | 1.35 (1.18-1.54) | |  | | 166 | 1.30 | 1.25 (1.04-1.50) | |
| ≥5 pain sites | 1895 | 310 | 1.64 | 1.49 (1.31-1.69) |  | 231 | 1.75 | 1.56 (1.34-1.81) | |  | | 126 | 1.45 | 1.36 (1.10-1.67) | |

CI, confidence interval; RR, risk ratio. ^a^ HADS-A ≥8 and/or HADS-D≥8; ^b^ HADS-A≥8 and any HADS-D; ^c^ HADS-D≥8 and any HADS-A

^*^Adjusted for age (continuous)

^†^ Multi-adjusted for age (continuous), sex (women, men), body mass index (continuous), smoking (never, former or occasional, current), alcohol consumption (no alcohol abuse, possible alcohol abuse), physical activity level (inactive, low activity, moderate activity, high activity)

Table S4. Joint effect of number of chronic pain sites and insomnia symptoms on risk of anxiety and depression at follow up (excluding people reporting physical pain (strong or very strong) over the last month among those without chronic pain)

| Chronic pain variables | No insomnia symptoms | | |  | | Any insomnia symptoms ^a^ | | | | |  | | Insomnia (ICSD-3) ^b^ | | | |
| --- | --- | --- | --- | --- | --- | --- | --- | --- | --- | --- | --- | --- | --- | --- | --- | --- |
|  | No. of persons | No. of cases | Multi-adjusted, RR^†^ (95% CI) | |  | | No. of persons | No. of cases | Multi-adjusted RR^†^ (95% CI) |  | | No. of persons | | No. of cases | Multi-adjusted RR^†^ (95% CI) |  |
|  |  |  |  |  |  | |  |  |  |  | |  |  |  |  |  |
| No chronic pain | 6901 | 631 | 1.00 (reference) | |  | 1319 | | 200 | 1.64 (1.41-1.90) |  | | 207 | | 36 | 1.78 (1.31-2.42) |  |
| 1-2 pain sites | 3063 | 312 | 1.12 (0.99-1.28) | |  | 952 | | 149 | 1.70 (1.44-2.01) |  | | 146 | | 21 | 1.49 (1.00-2.24) |  |
| 3-4 pain sites | 1826 | 223 | 1.33 (1.15-1.53) | |  | 834 | | 136 | 1.76 (1.48-2.09) |  | | 205 | | 42 | 2.17 (1.64-2.87) |  |
| ≥5 pain sites | 968 | 139 | 1.54 (1.29-1.83) | |  | 717 | | 123 | 1.83 (1.53-2.20) |  | | 210 | | 48 | 2.44 (1.88-3.20) |  |

CI, confidence interval; RR, risk ratio.

^a^ Any of the following symptoms: ‘difficulty falling asleep’, ‘difficulty maintaining sleep’, ‘waking up too early’, ‘daytime sleepiness’

^b^ At least one nighttime insomnia symptom (‘difficulty falling asleep’, ‘difficulty maintaining sleep’, or ‘waking up too early’) accompanied by daytime sleepiness

^†^ Multi-adjusted for age (continuous), sex (women, men), body mass index (continuous), smoking (never, former or occasional, current), alcohol consumption (no alcohol abuse, possible alcohol abuse), physical activity level (inactive, low activity, moderate activity, high activity)

Table S5. Risk of anxiety and/or depression at follow-up associated with number of chronic pain sites at baseline (excluding people reporting fibromyalgia)

| Chronic pain variables | Anxiety and/or depression ^a^ | | | |  | Anxiety ^b^ | | |  | | Depression ^c^ | | | |  |
| --- | --- | --- | --- | --- | --- | --- | --- | --- | --- | --- | --- | --- | --- | --- | --- |
|  | No. of persons | No. of cases | Age-adjusted RR^*^ | Multi-adjusted, RR^†^ (95% CI) |  | No. of cases | Age-adjusted RR^*^ | Multi-adjusted RR^†^ (95% CI) | |  | | No. of cases | Age-adjusted RR^*^ | Multi-adjusted RR^†^ (95% CI) | |
|  |  |  |  |  |  |  |  |  |  |  | |  |  |  |  |
| No chronic pain | 9351 | 955 | 1.00 | 1.00 (reference) |  | 724 | 1.00 | 1.00 (reference) | |  | | 390 | 1.00 | 1.00 (reference) | |
| 1-2 pain sites | 4120 | 475 | 1.14 | 1.13 (1.02-1.26) |  | 346 | 1.14 | 1.13 (1.00-1.28) | |  | | 236 | 1.32 | 1.30 (1.11-1.52) | |
| 3-4 pain sites | 2745 | 378 | 1.38 | 1.33 (1.19-1.49) |  | 282 | 1.44 | 1.36 (1.19-1.55) | |  | | 157 | 1.29 | 1.28 (1.07-1.54) | |
| ≥5 pain sites | 1549 | 246 | 1.60 | 1.52 (1.33-1.74) |  | 180 | 1.70 | 1.56 (1.33-1.83) | |  | | 104 | 1.48 | 1.47 (1.19-1.82) | |

CI, confidence interval; RR, risk ratio. ^a^ HADS-A ≥8 and/or HADS-D≥8; ^b^ HADS-A≥8 and any HADS-D; ^c^ HADS-D≥8 and any HADS-A

^*^Adjusted for age (continuous)

^†^ Multi-adjusted for age (continuous), sex (women, men), body mass index (continuous), smoking (never, former or occasional, current), alcohol consumption (no alcohol abuse, possible alcohol abuse), physical activity level (inactive, low activity, moderate activity, high activity)

Table S6. Joint effect of number of chronic pain sites and insomnia symptoms on risk of anxiety and depression at follow up (excluding people reporting fibromyalgia)

| Chronic pain variables | No insomnia symptoms | | |  | | Any insomnia symptoms ^a^ | | | | |  | | Insomnia (ICSD-3) ^b^ | | | |
| --- | --- | --- | --- | --- | --- | --- | --- | --- | --- | --- | --- | --- | --- | --- | --- | --- |
|  | No. of persons | No. of cases | Multi-adjusted, RR^†^ (95% CI) | |  | | No. of persons | No. of cases | Multi-adjusted RR^†^ (95% CI) |  | | No. of persons | | No. of cases | Multi-adjusted RR^†^ (95% CI) |  |
|  |  |  |  |  |  | |  |  |  |  | |  |  |  |  |  |
| No chronic pain | 7638 | 700 | 1.00 (reference) | |  | 1487 | | 217 | 1.57 (1.36-1.81) |  | | 226 | | 38 | 1.72 (1.28-2.32) |  |
| 1-2 pain sites | 3040 | 308 | 1.11 (0.98-1.26) | |  | 936 | | 146 | 1.69 (1.44-2.00) |  | | 144 | | 21 | 1.50 (1.00-2.25) |  |
| 3-4 pain sites | 1766 | 215 | 1.32 (1.14-1.53) | |  | 785 | | 123 | 1.69 (1.41-2.02) |  | | 194 | | 40 | 2.18 (1.64-2.90) |  |
| ≥5 pain sites | 831 | 116 | 1.50 (1.25-1.80) | |  | 565 | | 92 | 1.75 (1.43-2.15) |  | | 153 | | 38 | 2.67 (1.99-3.57) |  |

CI, confidence interval; RR, risk ratio.

^a^ Any of the following symptoms: ‘difficulty falling asleep’, ‘difficulty maintaining sleep’, ‘waking up too early’, ‘daytime sleepiness’

^b^ At least one nighttime insomnia symptom (‘difficulty falling asleep’, ‘difficulty maintaining sleep’, or ‘waking up too early’) accompanied by daytime sleepiness

^†^ Multi-adjusted for age (continuous), sex (women, men), body mass index (continuous), smoking (never, former or occasional, current), alcohol consumption (no alcohol abuse, possible alcohol abuse), physical activity level (inactive, low activity, moderate activity, high activity)

Table S7. Risk of anxiety and/or depression at follow-up associated with number of chronic pain sites at baseline adjusting for comorbidities

| Chronic pain variables | No. of persons | Anxiety and/or depression ^a^ | | |  | | Anxiety ^b^ | | |  | Depression ^c^ | | |
| --- | --- | --- | --- | --- | --- | --- | --- | --- | --- | --- | --- | --- | --- |
|  |  | No. of cases | Age-adjusted RR^*^ | Multi-adjusted, RR^†^ (95% CI) | |  | No. of cases | Age-adjusted RR^*^ | Multi-adjusted RR^†^ (95% CI) |  | No. of cases | Age-adjusted RR^*^ | Multi-adjusted RR^†^ (95% CI) |
|  |  |  |  |  |  |  |  |  |  |  |  |  |  |
| No chronic pain | 9380 | 962 | 1.00 | 1.00 (reference) | |  | 730 | 1.00 | 1.00 (reference) |  | 394 | 1.00 | 1.00 (reference) |
| 1-2 pain sites | 4161 | 482 | 1.14 | 1.15 (1.03-1.28) | |  | 352 | 1.14 | 1.15 (1.01-1.30) |  | 240 | 1.32 | 1.32 (1.13-1.56) |
| 3-4 pain sites | 2865 | 401 | 1.39 | 1.34 (1.19-1.50) | |  | 302 | 1.47 | 1.38 (1.21-1.59) |  | 166 | 1.30 | 1.28 (1.06-1.54) |
| ≥5 pain sites | 1895 | 310 | 1.64 | 1.57 (1.36-1.80) | |  | 231 | 1.76 | 1.69 (1.43-1.98) |  | 126 | 1.45 | 1.43 (1.14-1.79) |

CI, confidence interval; RR, risk ratio.

^a^ HADS-A ≥8 and/or HADS-D≥8; ^b^ HADS-A≥8 and any HADS-D; ^c^ HADS-D≥8 and any HADS-A

^*^Adjusted for age (continuous)

^†^ Multi-adjusted for age (continuous), sex (women, men), body mass index (continuous), smoking (never, former or occasional, current), alcohol consumption (no alcohol abuse, possible alcohol abuse), physical activity level (inactive, low activity, moderate activity, high activity), comorbidities (cardiovascular disease, diabetes, rheumatic/degenerative joint disease, cancer)

Table S8. Joint effect of number of chronic pain sites and insomnia symptoms on risk of anxiety and depression at follow up adjusting for comorbidities

| Chronic pain variables | No insomnia symptoms | | | |  | Sub-threshold insomnia ^a^ | | | |  | Insomnia (ICSD-3) ^b^ | | | |
| --- | --- | --- | --- | --- | --- | --- | --- | --- | --- | --- | --- | --- | --- | --- |
|  | No. of persons | No. of cases | Age-adjusted RR^*^ | Multi-adjusted, RR^†^ (95% CI) |  | No. of persons | No. of cases | Age-adjusted RR^*^ | Multi-adjusted RR^†^ (95% CI) |  | No. of persons | No. of cases | Age-adjusted RR^*^ | Multi-adjusted RR^†^ (95% CI) |
|  |  |  |  |  |  |  |  |  |  |  |  |  |  |  |
| No chronic pain | 7653 | 703 | 1.00 | 1.00 (reference) |  | 1497 | 219 | 1.60 | 1.55 (1.35-1.79) |  | 230 | 40 | 1.86 | 1.75 (1.31-2.35) |
| 1-2 pain sites | 3063 | 312 | 1.12 | 1.12 (0.98-1.27) |  | 952 | 149 | 1.73 | 1.68 (1.42-1.98) |  | 146 | 21 | 1.55 | 1.46 (0.98-2.19) |
| 3-4 pain sites | 1826 | 223 | 1.35 | 1.31 (1.13-1.52) |  | 834 | 136 | 1.82 | 1.73 (1.45-2.05) |  | 205 | 42 | 2.24 | 2.12 (1.61-2.81) |
| ≥5 pain sites | 968 | 139 | 1.60 | 1.52 (1.27-1.81) |  | 717 | 123 | 1.93 | 1.80 (1.49-2.18) |  | 210 | 48 | 2.56 | 2.37 (1.80-3.12) |

CI, confidence interval; RR, risk ratio.

^a^ At least one of the following symptoms: ‘difficulty falling asleep’, ‘difficulty maintaining sleep’, ‘waking up too early’, ‘daytime sleepiness’

^b^ At least one nighttime insomnia symptom (‘difficulty falling asleep’, ‘difficulty maintaining sleep’, or ‘waking up too early’) accompanied by daytime sleepiness

^†^ Multi-adjusted for age (continuous), sex (women, men), body mass index (continuous), smoking (never, former or occasional, current), alcohol consumption (no alcohol abuse, possible alcohol abuse), physical activity level (inactive, low activity, moderate activity, high activity), comorbidities (cardiovascular disease, diabetes, rheumatic/degenerative joint disease, cancer)

Table S9. Risk of anxiety and/or depression at follow-up associated with number of chronic pain sites at baseline stratified by insomnia subgroups

| Chronic pain variables | No insomnia symptoms | | | |  | Sub-threshold insomnia ^a^ | | | |  | Insomnia (ICSD-3) ^b^ | | | |
| --- | --- | --- | --- | --- | --- | --- | --- | --- | --- | --- | --- | --- | --- | --- |
|  | No. of persons | No. of cases | Age-adjusted RR^*^ | Multi-adjusted, RR^†^ (95% CI) |  | No. of persons | No. of cases | Age-adjusted RR^*^ | Multi-adjusted RR^†^ (95% CI) |  | No. of persons | No. of cases | Age-adjusted RR^*^ | Multi-adjusted RR^†^ (95% CI) |
|  |  |  |  |  |  |  |  |  |  |  |  |  |  |  |
| No chronic pain | 7653 | 703 | 1.00 | 1.00 (reference) |  | 1497 | 219 | 1.00 | 1.00 (reference) |  | 230 | 40 | 1.00 | 1.00 (reference) |
| 1-2 pain sites | 3063 | 312 | 1.12 | 1.11 (0.98-1.26) |  | 952 | 149 | 1.08 | 1.08 (0.89-1.30) |  | 146 | 21 | 0.84 | 0.83 (0.51-1.35) |
| 3-4 pain sites | 1826 | 223 | 1.35 | 1.31 (1.14-1.52) |  | 834 | 136 | 1.14 | 1.11 (0.91-1.35) |  | 205 | 42 | 1.21 | 1.20 (0.82-1.78) |
| ≥5 pain sites | 968 | 139 | 1.60 | 1.52 (1.28-1.81) |  | 717 | 123 | 1.20 | 1.15 (0.94-1.42) |  | 210 | 48 | 1.38 | 1.35 (0.93-1.99) |

CI, confidence interval; RR, risk ratio.

^a^ Any of the following symptoms: ‘difficulty falling asleep’, ‘difficulty maintaining sleep’, ‘waking up too early’, ‘daytime sleepiness’

^b^ At least one nighttime insomnia symptom (‘difficulty falling asleep’, ‘difficulty maintaining sleep’, or ‘waking up too early’) accompanied by daytime sleepiness

^†^ Multi-adjusted for age (continuous), sex (women, men), body mass index (continuous), smoking (never, former or occasional, current), alcohol consumption (no alcohol abuse, possible alcohol abuse), physical activity level (inactive, low activity, moderate activity, high activity)
